# Mast Cells Are a Reservoir of NLRP1 in Human Skin

**DOI:** 10.3390/ijms27093775

**Published:** 2026-04-23

**Authors:** Alexandra Dobre, Tudor Emanuel Fertig, Andrei Marian Niculae, Adelina Maria Cohn, Antoanela Curici, Razvan Theodor Andrei, Daciana Silvia Marta, Victor Eduard Peteu, Roua Gabriela Popescu, George Catalin Marinescu, Gabriela Turcu, Ana Maria Forsea, Daniela Adriana Ion, Mihaela Gherghiceanu, Roxana Ioana Nedelcu

**Affiliations:** 1 Faculty of Medicine, Carol Davila University of Medicine and Pharmacy, 020021 Bucharest, Romania; alexandra.dobre@drd.umfcd.ro (A.D.);; 2 Dermatology, Elias University Emergency Hospital, 011461 Bucharest, Romania; 3 Victor Babeş National Institute of Pathology, 050096 Bucharest, Romania; 4 Laboratoire National De Santé, 3555 Dudelange, Luxembourg; 5 Synevo Romania, 014192 Bucharest, Romania; 6 Colentina Clinical Hospital, 020125 Bucharest, Romania; 7 Faculty of Medicine, Titu Maiorescu University, 040051 Bucharest, Romania; 8 Asociația Independent Research, 012416 Bucharest, Romania; 9 Blue Screen SRL, 012416 Bucharest, Romania; 10 Derma 360 Clinic, 011273 Bucharest, Romania; 11 Faculty of Medicine, Health and Medical University Potsdam, 14471 Potsdam, Germany

**Keywords:** NLRP1, inflammasome, mast cells, autoinflammatory disease, multiple self-healing palmoplantar carcinoma, keratoacanthoma

## Abstract

NLRP1 is an inflammasome sensor protein expressed in barrier tissues of humans. Its activation in response to microbes or cellular stress triggers a cascade of molecular events, leading up to IL1β-driven inflammation and pyroptosis. Rare germline mutations of NLRP1 cause its persistent activation, resulting in autoinflammatory syndromes. Multiple self-healing palmoplantar carcinoma (MSPC) is one such syndrome, characterized by the appearance of recurrent keratoacanthomas (KAs) on the palms and soles. Here, we aimed to compare the subcellular localization of mutant NLRP1 in lesions from an MSPC patient to wild-type NLRP1 in non-MSPC-KAs and in skin from healthy donors. Using mass spectrometry, immunohistochemistry and immunoelectron tomography, we found that NLRP1 localized to mast cell granules in all MSPC lesions but also in healthy skin, a novel finding which implicates these cells in NLRP1-associated responses in human skin. Moreover, we found that mast cells expressing the A66V pathogenic variant of NLRP1 overpopulated MSPC-KAs, infiltrated the epidermis and degranulated, a behavior not seen in other lesions from this study. The released granules had the highest NLRP1 protein content and also contained NLRP3 and IL1β, suggesting the coexistence of inflammasome pathways within mast cells. Taken together, our findings propose cutaneous mast cells as a previously unrecognized NLRP1 reservoir in health and disease.

## 1. Introduction

NLRP1 (NLR family pyrin domain containing 1) is a cytosolic sensor protein with a major role in innate immunity. In response to pathogens or other stress signals, NLRP1 can participate in the formation of an intracellular protein complex termed the “inflammasome” [1]. This assembly involves the recruitment of the adapter protein ASC (apoptosis-associated speck-like protein containing a CARD), which facilitates the activation of caspase-1. Once activated, caspase-1 mediates the maturation and secretion of pro-inflammatory cytokines IL-1β and IL-18 and the induction of pyroptosis [2].

In humans, NLRP1 pathogenic variants can lead to a variety of clinical syndromes depending on the affected protein domain, mainly involving the skin, cornea and lungs [3,4,5,6]. Of these, multiple self-healing palmoplantar carcinoma (MSPC) is characterized by recurrent keratoacanthomas (KAs) that develop on palmoplantar skin and sporadically on other epithelia lacking hair follicles [7]. Responsible are either one of three germline heterozygous mutations (A54T, A66V, M77V) affecting the pyrin domain (PYD) of NLRP1, causing spontaneous self-oligomerization of the protein, aberrant inflammasome activation and subsequent proliferation of select keratinocytes [7].

Keratinocytes are known as the main NLRP1 reservoir in human skin [7,8,9,10]; however, granulocytes, lymphocytes, macrophages and dendritic cells also express NLRP1 [11]. RNA-seq data from the Human Protein Atlas indicates basophils as having the highest potential for NLRP1 protein expression among immune cells [12,13].

Mast cells (MCs) are tissue-resident immune cells of hematopoietic origin, which differentiate from a basophil–MC common progenitor. MCs are known to contain NLRP3 within secretory granules and can produce IL1β following NLRP3 inflammasome activation [14,15]. Mutations of NLRP3 cause it to become constitutively activated in MCs, initiating overproduction of IL1β. This contributes to a spectrum of clinical entities named cryopyrin-associated periodic syndromes (CAPSs), in which the skin, joints and central nervous system can be affected [14,16]. While MCs from lung tissue were recently shown to express *NLRP1* transcripts [17], the presence of the functional protein in MCs remains uncharacterized.

We report for the first time at the protein level that skin MCs can harbor NLRP1 within granules. We further show degranulation of MCs in three temporally distinct KAs of an MSPC patient with NLRP1^A66V^ and suggest that release of granules containing this hyperactive NLRP1 variant contributes to the pathogenesis of KAs in this syndrome.

## 2. Results

### 2.1. Different Skin Lesions from an MSPC Patient Have Divergent Patterns of Inflammasome Activation

**Figure 1:**
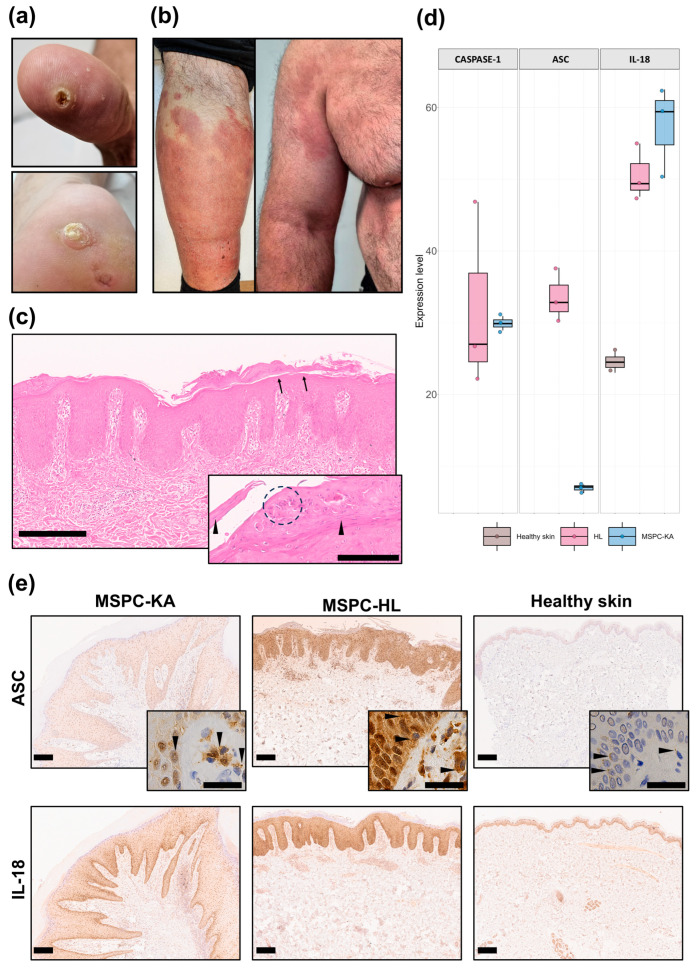
Inflammasome activation is different between two types of skin lesions from a patient with multiple self-healing palmoplantar carcinoma (MSPC). (**a**) The MSPC patient presented keratoacanthomas (KAs) at different stages of evolution on the palmar and plantar surfaces. (**b**) The MSPC patient suddenly developed a widespread eruption of well-defined erythemato-squamous plaques. (**c**) Hematoxylin-and-eosin staining (HE, 10× and 20× inset) of an abdominal lesion from the MSPC patient, showed psoriasiform acanthosis with elongation of the rete ridges, thinned suprapapillary plates, hypervascular dermal papillae, neutrophil clusters (circle) in the parakeratotic stratum corneum (arrow heads) and hypogranulosis (arrows); scale bars are 300 and 100 µm for inset. (**d**) Mass spectrometry (MS) showing higher protein expression levels for caspase-1, apoptosis-associated speck-like protein containing a CARD (ASC) and interleukin-18 (IL-18) in the abdominal hyperkeratotic inflammatory lesion (HL) and MSPC-KAs compared to healthy skin (*n* = 1 biopsy for each). ASC levels were highest in MSPC-HL but were below detection levels in healthy skin. (**e**) Spatial distribution of inflammasome-associated proteins ASC (top row and insets) and IL-18 (bottom row) in MSPC lesions and healthy skin. The epidermis of MSPC-HL was strongly positive for ASC and IL-18, which appeared uniformly distributed throughout the cell layers. In MSPC-KAs, both proteins localized to cell clusters in the dermis, but epidermal staining was weaker when compared to MSPC-HL. Granular material suggestive of ASC speck formation is indicated by arrow heads in insets. Scale bars are 200 µm for all images in (**e**).

A 49-year-old male with a history of multiple central hyperkeratotic lesions on the palms and soles (Figure 1a) in the context of MSPC with NLRP1^A66V^ presented with symmetrically distributed and well-defined erythematous–squamous plaques, some with erosions and crusting. These initially appeared on the dorsal feet and later spread to the extremities, trunk and head (Figure 1b). An infection was ruled out because of unresponsiveness to antibiotics/antifungals and negative PAS staining. Although histopathology suggested psoriasis (Figure 1c and inset), allergic contact dermatitis was also suspected due to (a) positive patch tests to lanolin alcohol and nickel(II) sulfate hexahydrate, (b) good response of lesions to oral corticotherapy and lanolin-free moisturizers, and (c) lack of recurrence after stopping corticotherapy. Due to the presence of these overlapping features and the atypical development and clinical presentation of the eruption, these plaques were interpreted as atypical hyperkeratotic inflammatory lesions (HLs).

MSPC patients characteristically develop palmoplantar KAs, and it remains unclear why KAs fail to form on other anatomical regions. The sudden appearance of these extensive HLs in this MSPC patient presented a unique opportunity to compare their lesional microenvironment to that of different plantar KAs from the same individual (biopsied before, concomitantly and after MSPC-HL diagnosis) in an attempt to isolate the etiology of MSPC-KAs.

We first asked whether there are differences in inflammasome activation between the two lesion types and we employed MS and IHC to investigate protein expression. As expected, both MSPC-KAs (*n* = 1 biopsy) and MSPC-HL (*n* = 1 biopsy) showed higher abundance of key inflammasome-associated proteins compared to healthy skin (*n* = 1 biopsy, Figure 1d,e). MS analyses revealed that whereas caspase-1 and IL-18 had similar levels of expression in the two MSPC lesions, ASC was significantly overexpressed in MSPC-HL (Figure 1d). This may indicate higher priming of inflammasome pathways and/or accumulation and persistence of stable ASC-based supramolecular complexes in MSPC-HL tissue.

These complexes (known as “specks”) are a hallmark of inflammasome activation and are readily detectable by light microscopy as discrete puncta in the nucleus and cytosol [18,19]. Consistent with MS data, the strongest immunoreactivity for ASC was in the MSPC-HL epidermis (*n* = 1 biopsy). Here, and to a lesser extent in the subjacent dermis, cells exhibited a dispersed punctate pattern indicative of different stages of speck assembly (Figure 1e, top row and inset). In MSPC-KA lesions (*n* = 3 biopsies), ASC also had a strong, granular signal associated with dermal cell clusters, contrasting with an unexpectedly weak staining of keratinocytes in the overlying epidermis (Figure 1e, top row and inset). The differences in staining intensity for IL-18 were less striking but still favored the epidermis of MSPC-HL, where it appeared uniformly distributed across cell layers. In MSPC-KAs, IL-18 also concentrated to cell clusters in the dermis and in the basal layer of the epidermis (Figure 1e, bottom row). These results suggested the existence of different hotspots for inflammasome activation in the two MSPC lesions, potentially involving site-specific cell populations and pathogenic mechanisms.

### 2.2. MCs Accumulate in MSPC-KAs

Given that both MSPC-KAs and MSPC-HL showed increased inflammasome activity but divergent ASC staining patterns, we hypothesized that a cell-specific distribution of NLRP1^A66V^ may, at least in part, explain the formation of characteristic KAs on palmoplantar skin but not on skin from other anatomical regions.

**Figure 2:**
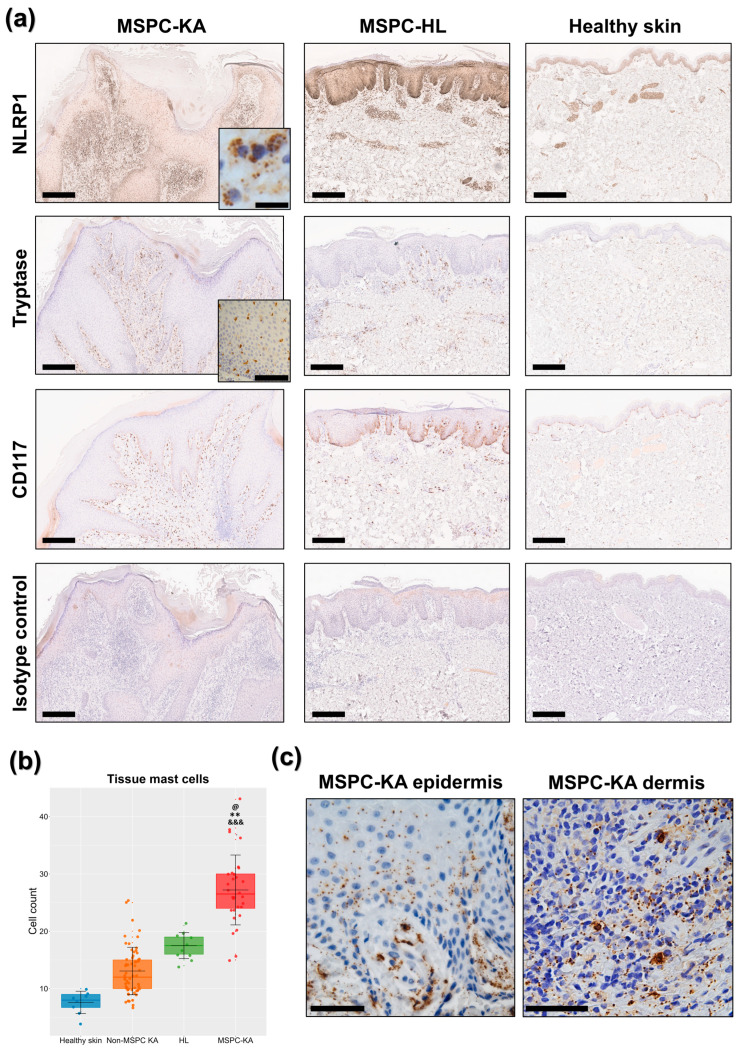
Immunohistochemistry of keratoacanthomas (KAs) in multiple self-healing palmoplantar carcinoma (MSPC). (**a**) In MSPC-KAs, NLRP1^A66V^ was more highly expressed in the dermis and was concentrated in cell granules (top row inset, scale bar = 10 µm). In an atypical hyperkeratotic inflammatory lesion (HL), NLRP1^A66V^ was overrepresented in the epidermis. CD117 and tryptase staining demonstrated more mast cells (MCs) in MSPC-KAs, including epidermal infiltration (second row inset, scale bar = 100 µm). The isotype control did not bind. Scale bars = 300 µm. (**b**) Boxplots show the distribution of MCs per group. Statistical comparisons were performed on mean MC counts per biopsy, as identified by tryptase staining. Biological sample size (distinct biopsies): healthy skin (*n* = 1), HL (*n* = 1), non-MSPC-KAs (*n* = 6), MSPC-KAs (*n* = 3). Statistical significance is shown against healthy skin—*, HL—@, and the non-MSPC-KAs group—&, where @ *p* < 0.05; ** *p* < 0.01; &&& *p* < 0.001. (**c**) MCs in MSPC-KAs were degranulated, with granular material dispersed in the epidermis and dermis. Scale bars = 50 µm.

In IHC, NLRP1 distribution and staining intensity mimicked our spatial data on ASC. Keratinocytes from MSPC-KAs (*n* = 3 biopsies) were only discretely positive for NLRP1, with a nucleo-cytoplasmic staining pattern and the intensity gradually decreasing towards the upper epidermal layers (Figure 2a). Contrary to previous reports [7], the NLRP1^A66V^ signal was more intense in the dermis than the epidermis, with specific cell populations presenting a granular distribution within the cytoplasm (Figure 2a, top row inset). Inversely, NLRP1^A66V^ expression was highest in the epidermis of the MSPC-HL sample (*n* = 1) but comparatively reduced in the dermis, mainly clustering around blood vessels (Figure 2a). Healthy skin (*n* = 1 biopsy) showed the least NLRP1 staining intensity in both the epidermis and dermis (Figure 2a), although the granular distribution persisted in cells surrounding blood vessels. Positivity of immune cell granules for NLRP1 was confirmed on a human lymph node specimen (Supplementary Figure S1a), whereas the isotype control did not bind (Figure 2a).

To investigate whether some of the numerous NLRP1-positive cells in the MSPC-KA dermis were MCs, we stained using tryptase and CD117/c-kit (Figure 2a), and then tryptase-positive cells were counted on ten consecutive high-power fields for each biopsy. Indeed, KAs from the MSPC patient had significant MC accumulation (mean 27.2/mm^2^, *n* = 3 biopsies), above MSPC-HL (17.5/mm^2^, *n* = 1 biopsy) or healthy skin (7.6/mm^2^, *n* = 1 biopsy) (Figure 2b). In MSPC-KAs but not MSPC-HL, MCs frequently infiltrated epidermal layers (Figure 2a, second row inset).

Under higher magnification, we further observed that MCs in MSPC-KAs, but not MSPC-HL, were degranulated. Tryptase-positive granular material was widely distributed within connective tissue and taken up in the epidermis, up to the spinous layer (Figure 2c). To verify if these are common features of all KAs, we used tryptase staining on six KAs from patients not diagnosed with MSPC (Supplementary Figure S1b). As expected [20], these presented MCs in higher numbers (mean 13/mm^2^, *n* = 6 biopsies) than healthy skin; however, they were below counts in MSPC-KAs and even MSPC-HL (Figure 2b). Importantly, we could not identify MC epidermal infiltration nor MC degranulation, indicating their distinct contribution in MSPC-KA pathogenesis.

The MSPC patient was negative for D816V KIT in whole blood and had normal serum tryptase (<11.4 µg/L), limiting the possibility of MC neoplastic transformation.

Taken together, these results suggest that plantar KAs from an MSPC patient with the NLRP1^A66V^ pathogenic variant have a specific cellular landscape, dominated by high numbers of degranulating MCs, which can infiltrate the epidermis.

### 2.3. NLRP1 Localizes to MC Granules

**Figure 3:**
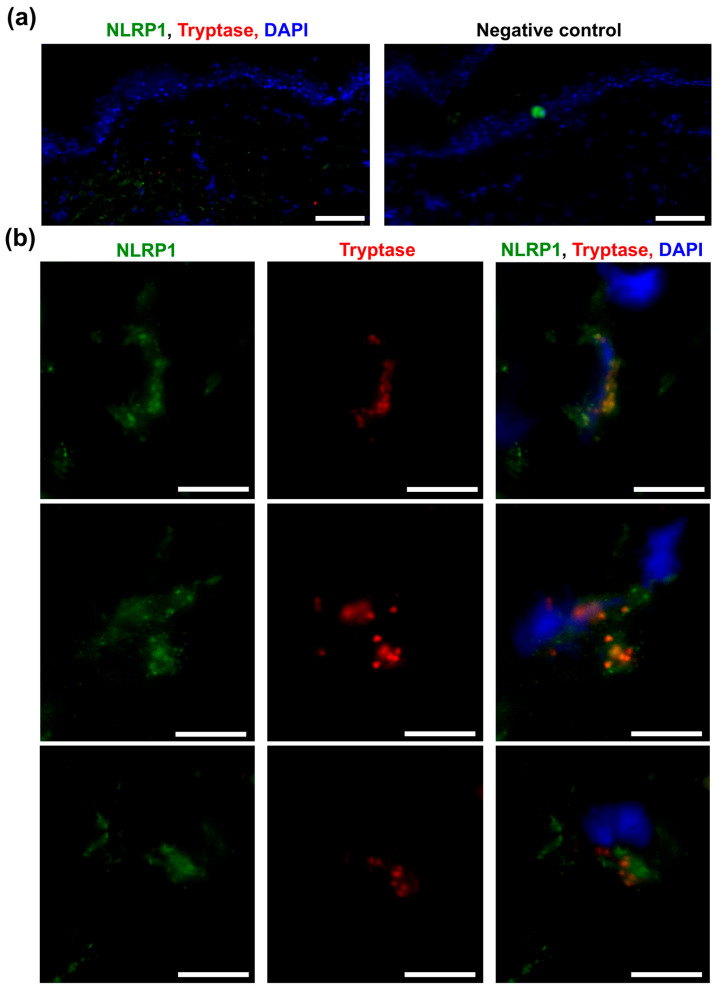
NLRP1 localizes to mast cells (MCs) in healthy human skin. (**a**) The NLRP1 signal (green) was strongest in the dermis from the fresh frozen skin of a healthy donor (left) but was absent when the primary antibody was omitted (right). Nuclei were stained with DAPI (blue). Scale bars = 100 µm. (**b**) Representative high magnification (100× objective) images of three dermal MCs from healthy human skin, showing colocalization of NLRP1 (green) and tryptase (red) in MC granules. The MC from the first row is represented in 3D in Supplementary Video S1. Nuclei were stained with DAPI (blue). Scale bars = 10 µm.

To verify whether MCs can represent a reservoir of NLRP1 in skin, we first used immunofluorescence on frozen sections from the skin of a healthy donor (*n* = 1 biopsy). Consistent with IHC data, at low magnification, the NLRP1 signal was discrete but stronger in the dermis than the epidermis (Figure 3a). At higher magnification, however, NLRP1 was observed within MCs and showed clear colocalization with tryptase within cytoplasmic granules (Figure 3b, Supplementary Video S1), suggesting that NLRP1 is likely an endogenous constituent of the skin MC proteome.

**Figure 4:**
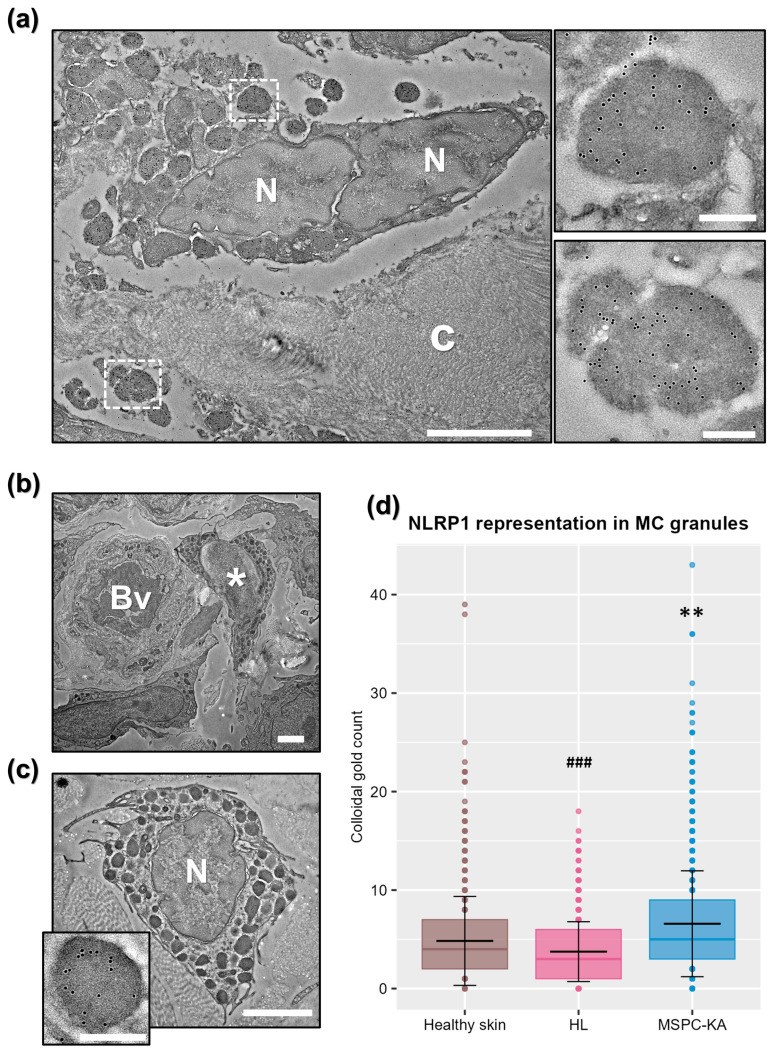
NLRP1 is ubiquitous in skin mast cell (MC) granules. (**a**) MCs in keratoacanthomas (KAs) from a multiple self-healing palmoplantar carcinoma (MSPC) patient were degranulated. Scale bar = 2 µm. c—collagen, N—nucleus. MC granules in MSPC-KAs presented NLRP1^A66V^ accumulation, as exemplified by higher magnifications of boxed areas. Gold labeling is seen as black dots. Scale bars = 250 nm. (**b**) MCs from an atypical hyperkeratotic inflammatory lesion (HL) were morphologically normal (*) and clustered around blood vessels (Bv). Scale bar = 2 µm. (**c**) Normal MC in healthy skin presenting NLRP1 in granules (inset, scale bar = 250 nm). Scale bar = 2 µm. (**d**) Boxplots show the gold-conjugated antibody distribution within granules, across all MCs, in each sample. Each gold particle corresponded to a NLRP1 target. Statistical comparisons were performed across MC measurements using one-way ANOVA followed by Tukey’s post hoc test. Group sizes were as follows: healthy skin (*n* = 31), HLs (*n* = 19), and MSPC-KAs (*n* = 34). Statistical significance is shown against healthy skin—* and MSPC-KAs—#, where ** *p* < 0.01; ### *p* < 0.001.

For more precise subcellular localization of NLRP1, we then employed immunoelectron microscopy (IEM). Morphologically, most MCs from MSPC-KAs (*n* = 1 biopsy) were degranulated, ranging from having empty granules and granules fused with the plasma membrane to complete fragmentation of cells, with release of granular complexes in the extracellular space (Figure 4a). By contrast, MCs from MSPC-HL (*n* = 1 biopsy) and healthy skin (*n* = 1 biopsy) had a typical appearance (intact granule architecture, presence of plasma membrane folds) and localized in the proximity of blood vessels (Figure 4b,c).

Using two different commercially available anti-NLRP1 antibodies, we then confirmed that NLRP1 accumulates in the granules of MCs from both lesional and healthy skin, regardless of granule morphology and degranulation status of the cell, further indicating that NLRP1 is ubiquitous to skin MCs (Figure 4a,c and inset). By contrast, an isotype control did not bind (Supplementary Figure S2a). Next, we asked whether there are differences in MC NLRP1 content between samples, so we manually counted and averaged colloidal gold per granule under identical immunolabeling conditions. In this context, this method of quantification is more precise than Western blotting of tissue lysates, as it measures protein content in the subcellular compartment of interest, unbiased by overall representation of MCs in tissue. We found that NLRP1^A66V^ from MSPC-KAs was overrepresented compared to NLRP1^A66V^ in MSPC-HL or wild-type NLRP1 in healthy skin (Figure 4d), suggesting that MCs may serve as a reservoir of hyper-reactive NLRP1^A66V^ during MSPC-KA pathogenesis.

In accordance with IHC, NLRP1 was localized in keratinocytes in all samples; however, it unexpectedly clustered around intermediate filaments and desmosomes (Supplementary Figure S2b).

### 2.4. NLRP1 and NLRP3 Can Colocalize in MC Granules

**Figure 5:**
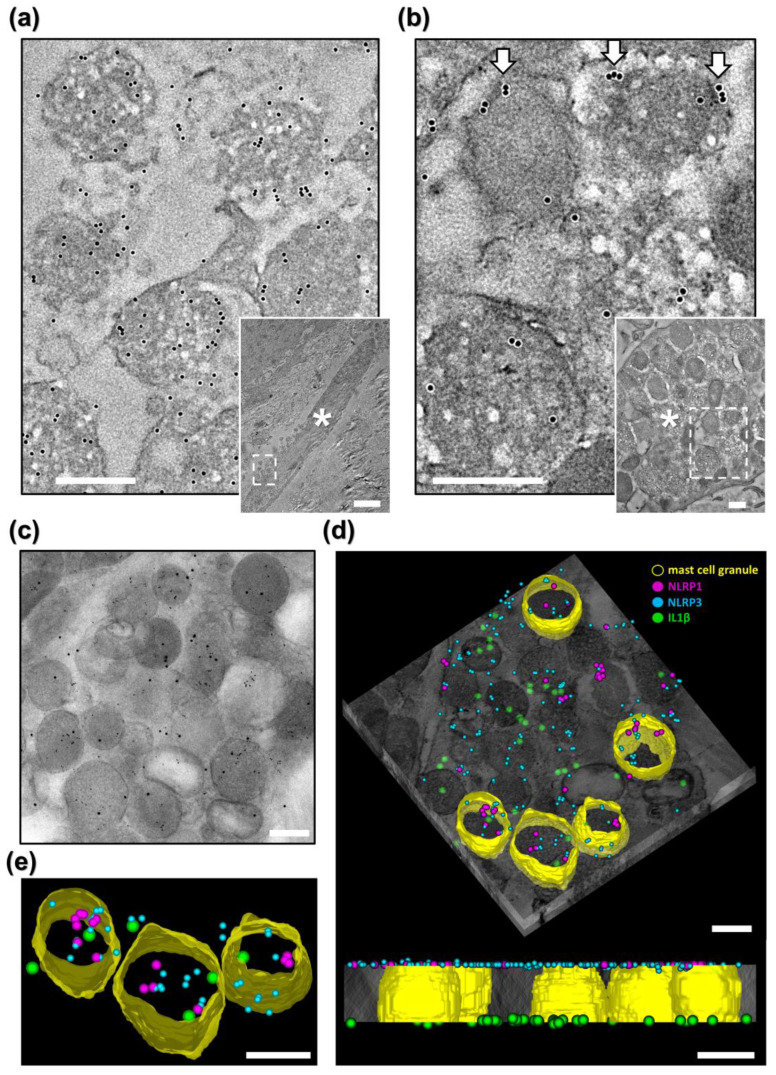
NLRP1^A66V^, NLRP3 and IL1β colocalize to mast cell (MC) granules in an MSPC keratoacanthoma. (**a**) High magnification of box in inset, confirming pro-IL1β/IL1β in granular material released by an MC (*). (**b**) High magnification of box in inset, showing MCs (*) containing NLRP3, which frequently localized to the periphery of granules (arrows). (**c**) Projection from tomographic tilt series, showing colocalization of gold labels (NLRP1^A66V^, NLRP3, IL1β) in MC granules. (**d**, **top**) Reconstructed tomogram with superimposed contours of five MC granules (yellow), presenting colocalization of NLRP1^A66V^ (magenta), NLRP3 (turquoise) and IL1β (green). (**d**, **bottom**) Side view showing distribution of labels to each side of the imaged resin section. (**e**) Detail of segmented MC granules. Scale bar for inset (**a**) = 2.5 µm, all others = 300 nm.

MCs are known to contain all components of the NLRP3 inflammasome and to secrete IL1β in an NLRP3-dependent manner [14,15]. Given our finding that NLRP1^A66V^ was present in MC granules from the MSPC patient, we were curious whether it spatially overlapped with NLRP3 and if it could therefore share elements of the inflammasome machinery during activation. First, we labeled MSPC-KA (*n* = 1 biopsy) against pro-IL1β/IL1β (hereafter IL1β) and found it to be well represented in both intracellular MC granules and in MC-derived extracellular granular material (Figure 5a and inset). This demonstrated that MCs in MSPC-KAs secrete this pro-inflammatory cytokine in the extracellular space during degranulation.

Next, we showed that some MC granules from MSPC-KA also contained NLRP3 (Figure 5b and inset) and released it during degranulation. NLRP3 was frequently localized at the periphery of cytoplasmic granules and at the plasma membrane in degranulating MCs (Figure 5b), in agreement with a recently published study showing a role for NLRP3 in granule exocytosis [21]. Electron tomography on double-labeled sections revealed that NLRP1 and NLRP3 can colocalize to some, but not all, MC granules (Supplementary Figure S2c,d, Supplementary Video S2), raising the possibility of compartmentalization of inflammasome pathways within MCs. Finally, triple-labeling electron tomography revealed that both sensor proteins can colocalize with IL1β in both intra- and extracellular MC granules (Figure 5c–e, Supplementary Video S3), suggesting that multiple inflammasome sensor proteins can simultaneously inhabit MC granules and may act in tandem during activation of MCs in MSPC-KAs.

## 3. Discussion

Here, we showed that skin MCs can act as a reservoir for NLRP1, a critical but lesser known inflammasome sensor protein. Although novel, this finding is not completely surprising, as MCs express pattern recognition receptors and harbor all components of the NLRP3 inflammasome pathway [15], some of which are also required for NLRP1 inflammasome assembly.

Further, our data indicated that MCs may play a role in the pathogenesis of KAs in an MSPC patient with NLRP1^A66V^. In three different plantar lesions, separated from initial diagnosis by 1, 8 and 10 years, MCs infiltrated the epidermis, and NLRP1^A66V^ was released in the dermis and epidermis through widespread MC degranulation. These features were conspicuously absent in an HL specimen from the abdominal skin of the same MSPC patient and from KAs from six non-MSPC patients.

The activation of MCs in MSPC-KAs from this patient may be a response to NLRP1-inflammasome activation in local keratinocytes. However, this contrasts with two of our key observations: (1) that the highest intensity of ASC and NLRP1 staining was in the MSPC-HL epidermis, where keratinocytes also express the hyperactive NLRP1^A66V^ yet do not form KAs, and (2) that compared to healthy skin, MSPC-HL presented high numbers of MCs prone to activation by keratinocyte inflammatory cues, but these MCs had normal morphology.

Alternatively, the particular MC behavior in the plantar skin of this MSPC patient may be the result of intrinsic NLRP1–inflammasome activation. Activation of select MC clusters and release of granular material within the epidermis could induce keratinocyte inflammatory responses, increasing MC recruitment within the lesion.

In support of this hypothesis, we showed that MCs and MC granular material contacted keratinocytes of the basal and spinous layers of MSPC-KAs. The granules had a higher NLRP1 content than equivalents in MSPC-HL and healthy skin and also harbored NLRP3 and IL1β. The latter can be converted to its active form within intact granules via the NLRP1 and NLRP3 inflammasome pathways but also in the extracellular environment by endogenous proteases [21,22]. Similarly, ASC and mutant NLRP3 particles have been shown by others to retain pro-inflammatory activity after being released from pyroptotic macrophages [23].

This study has three important limitations. First, in absence of functional studies, we cannot definitively ascertain whether MC degranulation is a prerequisite for MSPC-KA formation or if it is a local response mechanism which merely modulates lesion development. As murine NLRP1 lacks the PYD domain [7], an in vitro experiment would be required, involving co-culture of human keratinocytes with MCs expressing mutant NLRP1. Second, because we could only include a single MSPC patient carrying the NLRP1^A66V^ pathogenic variant, we are unable to extrapolate our findings to other MSPC variants. This is further hindered by the rarity of these diseases; of the five MSPC families reported worldwide [24], only one other harbors the A66V mutation [25]. Last, the lesions which we analyzed and compared in this study were obtained from different anatomical sites, which may have confounded the results.

In summary, we showed for the first time that NLRP1 is a component of skin MC granules and can colocalize with NLRP3 and IL1β, suggesting that MCs have more avenues for inflammasome activation than previously known. We also showed that when MCs from plantar skin express NLRP1^A66V^, they accumulate, infiltrate the epidermis and degranulate, possibly contributing to the pathogenesis of KAs in this variant of MSPC.

## 4. Materials and Methods

### 4.1. Patients and Samples

All subjects gave written informed consent prior to biopsy. The MSPC patient included here was diagnosed and genetically characterized in a previous study [7]. Four samples were collected from this patient: three different keratoacanthomas (KAs) from plantar regions (in 2016, 2023 and 2025) and a sample from an atypical hyperkeratotic inflammatory lesion (HL) on the abdomen (in 2023). The primary clinical distinction when performing sample collection was the presence of a central keratotic plug and nodular appearance of KAs, which were absent in the flatter, more diffuse HL specimen. Biopsies were also collected from three clinically healthy volunteers: two from the elbow region and one from the abdomen. The study included 6 archived formalin-fixed paraffin-embedded (FFPE) KA specimens from different patients with no clinical history of MSPC (non-MSPC-KAs). Human lymph node and tonsil FFPE specimens were included as NLRP1- and ASC-positive controls. All patients and archived FFPE specimens are listed in Supplementary Table S1. This study was performed in conformity with the Declaration of Helsinki and received approval from the ethics committees at Victor Babeș National Institute of Pathology and Elias University Emergency Hospital, Bucharest, Romania.

### 4.2. Light Microscopy and Immunohistochemistry

Hematoxylin–eosin analysis was done by a routine protocol. Immunohistochemistry (IHC) with the primary antibody of interest (Supplementary Table S2) was done using an Autostainer Link 48, following the manufacturer’s specifications (Dako, Agilent Technology, Santa Clara, CA, USA). An isotype control and omission of primary antibody were used to verify non-specific binding. Digital slides were generated using an automated slide scanner (Aperio AT2, Leica Biosystems, Vista, CA, USA). Images were processed by Contrast Limited Adaptive Histogram Equalization (CLAHE) in Fiji/ImageJ v1.54p [26], using the same settings for each type of staining.

### 4.3. Immunofluorescence

Fresh frozen skin tissue from a healthy donor (Supplementary Table S1) was sectioned using a cryostat (CM 1510S, Leica Microsystems, Wetzlar, Germany) at a 5 µm thickness. Primary antibodies against NLRP1 and tryptase (rabbit and mouse, Supplementary Table S2) were added simultaneously to the sections and incubated for 90 min at room temperature. Then, sections were washed and blocked for 60 min (905.002, Aurion, Wageningen, The Netherlands) before adding secondary antibodies at a 1:350 µL dilution: goat anti-rabbit IgG H&L Alexa Fluor^®^ 488 (ab150077, Abcam, Cambridge, UK) and goat anti-mouse IgG H&L Alexa Fluor^®^ 647 (ab150115, Abcam, UK). Negative controls had primary antibodies omitted. Mounting was done with ProLong™ antifade with DAPI (Invitrogen, Thermo Fisher Scientific, Waltham, MA, USA). Imaging and 3D rendering of image stacks were done on a TCS SP8 system (Leica Microsystems, Germany) using manufacturer-supplied software (LAS X v1.1, Leica Microsystems, Germany).

### 4.4. Mass Spectrometry

Biopsies were collected in cold PBS, and then 50 mg from each sample was disrupted in a Mini-Beadbeater (Biospec, Bartlesville, OK, USA). Samples were then centrifuged for 20 min at 18,000× *g*, and the protein concentration from the supernatant was quantified by a Bradford assay. A total of 50 μg of protein containing supernatant was diluted with urea buffer to the final volume of 40 μL, followed by the addition of 25 μL of 100 mM DTT and incubation at room temperature for 60 min. The sample was then alkylated with 26.25 μL of 300 mM iodoacetamide and kept in the dark at room temperature for an additional 90 min followed by a quenching reaction with 50 μL of DTT 100 mM at room temperature for 60 min. The reaction mix was finally diluted to 500 μL with 50 mM ammonium bicarbonate, followed by addition of trypsin (1 μg/μL; Trypsin Gold, V528A, Promega, Madison, WI, USA) at a 1:50 enzyme-to-substrate ratio, with incubation at 37 °C for 16 h. The reaction was stopped by adding 3 μL of formic acid. Peptides were cleaned up using solid-phase extraction (MonoSpin C18, GL Sciences, Fukushima, Japan), dried at speed-vac and rehydrated to 2 μg/μL using 5% ACN + 0.1% HCOOH. Peptide concentration was measured using NanoDrop 1000 (Thermo Fisher Scientific, USA). From each sample, 5 μg samples of peptides were loaded into a nanoACQUITY UPLC system (Waters, Milford, MA, USA) and separated using an Eksigent 5C18-CL-120 (300 μM ID, 150 mm length) column coupled with a TripleTOF 5600+ mass spectrometer (both AB Sciex, Framingham, MA, USA). Peptide samples (5 μg) were injected using Solvent A (0.1% formic acid) and separated on a 5–90% gradient of Solvent B (0.1% formic acid in acetonitrile) over 90 min at a flow rate of 5 μL/min, with the column maintained at 55 °C. Mass spectrometric (MS) analysis was carried out using electrospray ionization in positive mode, with a spray voltage of 5500 V and a source temperature of 200 °C. Data acquisition was performed on a TripleTOF 5600+ system in DIA SWATH-MS mode, with 64 variable isolation windows. The MS1 survey scans covered an *m*/*z* range of 400–1250, while MS2 spectra were collected in high-sensitivity mode from 100 to 2000 *m*/*z*. The accumulation period was set to 0.049 s, and ions were scanned within 55 ms windows in high-sensitivity mode, giving a total cycle time of 3.5 s. For each sample (biopsy), three technical LC–MS/MS replicate runs were performed. Peptide and protein identification were performed using PeakView 2.2, Skyline 22.2.0.255 and DIA-NN 1.9. MS data was deposited into the ProteomeXchange Consortium via the PRIDE partner repository, with the dataset identifier PXD073302 [27].

### 4.5. Immunoelectron Microscopy

Biopsies were fixed in a solution comprising 4% paraformaldehyde, 0.5% glutaraldehyde, and 1.4% sucrose (pH 7.4) and then embedded in London Resin White (LR White, Agar Scientific, Rotherham, UK). Ultrathin sections were mounted on formvar- and carbon-coated nickel grids and then incubated with the primary antibody of interest (Supplementary Table S2) and a gold-conjugated secondary antibody at 1:50 dilution (Aurion, The Netherlands). Negative controls had primary antibodies omitted, and an isotype control was used to exclude nonspecific binding to MC Fc receptors. A blocking solution for goat antibodies was used to reduce nonspecific binding (905.002, Aurion, The Netherlands). Imaging was done using a 4 × 4k Ceta camera on a Talos 200C transmission electron microscope (Thermo Fisher Scientific, USA).

### 4.6. Immunoelectron Tomography

LR White sections (300 nm) were adhered to nickel grids precoated with 0.01% poly-L-lysine and without formvar or carbon. For double-labeling, grids were incubated on each side, consecutively, with two primary antibodies raised in different species (mouse and rabbit, Supplementary Table S2). For triple-labeling, grids were incubated on one side with a mixture of two antibodies raised in different species (mouse and rabbit, Supplementary Table S2), and then either a rabbit or mouse primary antibody was used on the other side. Secondary antibodies had different colloidal gold diameters for each target (6, 10 and 15 nm, Aurion, The Netherlands). Absence of cross-contamination between section faces was verified by immunolabeling control grids on one side, while the other side was incubated with PBS. Acquisition of single-axis tilt series and tomographical reconstruction and segmentation in IMOD-eTomo v5.1.3 [28] were done as described elsewhere [29].

### 4.7. Data Analyses

Quantification of mast cells (MCs) was performed on tissue sections stained for tryptase. For each biopsy, tryptase-positive cells were manually counted in ten consecutive high-power fields (40× objective), starting from a hotspot. Counts were used to describe within-section variation in MC abundance. The biological sample size (distinct biopsies) was as follows: healthy skin (*n* = 1), HL (*n* = 1), non-MSPC-KAs (*n* = 6), and MSPC-KAs (*n* = 3).

To quantify the NLRP1 content in MC granules, MCs were identified on randomly selected ultrathin sections from MSPC biopsies (KA, *n* = 1 and HL, *n* = 1) and a healthy skin biopsy (*n* = 1), immunolabeled under identical conditions. Entire MCs were imaged, and colloidal gold particles were counted within all granules of each cell. These counts were used to describe the subcellular distribution of NLRP1. Analyzed MCs were considered biological replicates: healthy skin (*n* = 31), HL (*n* = 19), MSPC-KAs (*n* = 34).

Statistical analyses were performed on biologically independent units (mean counts per biopsy or per MC) using one-way ANOVA with Tukey’s post hoc test. Although statistical tests used only independent replicates, data are shown at the MC or colloidal-gold level to illustrate within-sample variation. Graphical representations were generated using R 4.4.1.

## Data Availability

Immunohistochemistry and transmission electron microscopy data other than presented in the article can be shared upon reasonable request to the corresponding author. The mass spectrometry proteomics data have been deposited into the ProteomeXchange Consortium via the PRIDE partner repository, with the dataset identifier PXD073302.

## Supplementary Materials

The following supporting information can be downloaded at: https://www.mdpi.com/article/10.3390/ijms27093775/s1.

## Abbreviations

The following abbreviations are used in this manuscript:

ACN	Acetonitrile
ASC	Apoptosis-associated speck-like protein containing a CARD
BSA	Bovine serum albumin
CAPS	Cryopyrin-associated periodic syndrome
CLAHE	Contrast-limited adaptive histogram equalization
DIA	Data independent acquisition
DTT	Dithiothreitol
FFPE	Formalin-fixed paraffin-embedded
HL	Atypical hyperkeratotic inflammatory lesions
IEM	Immunoelectron microscopy
IL1β	Interleukin-1 beta
IL18	Interleukin 18
KA	Keratoacanthoma
LR	London resin
NLR	Nucleotide-binding domain, leucine-rich repeat
NLRP1	NLR family pyrin domain containing 1
NLRP3	NLR family pyrin domain containing 3
MC	Mast cell
MS	Mass spectrometry
MSPC	Multiple self-healing palmoplantar carcinoma
PAS	Periodic acid–Schiff
PBS	Phosphate-buffered saline
PYD	Pyrin domain
RNA-seq	Ribonucleic acid sequencing
SWATH	Sequential window acquisition of full theoretical mass spectra
