# Supplementary material for: Mast Cells Are a Reservoir of NLRP1 in Human Skin
Source: Int J Mol Sci. 2026 Apr 23;27(9):3775. doi: 10.3390/ijms27093775 (PMC13164440; doi:10.3390/ijms27093775)

Table S1. Demographic data of tissue donors

Patient	Patient data		Lesion type	Number of biopsies / archived samples	Technique
	Sex	Age at biopsy			
1	M	50	Non-MSPC KA	1	IHC
2	F	83	Non-MSPC KA	1	IHC
3	M	77	Non-MSPC KA	1	IHC
4	F	59	Non-MSPC KA	1	IHC
5	M	62	Non-MSPC KA	1	IHC
6	F	88	Non-MSPC KA	1	IHC
7a	M	40	MSPC-KA	1	IHC
7b	M	47	MSPC-KA	1	IHC, IEM, IET
7c	M	47	MSPC-HL	1	IHC, MS, IEM
7d	M	49	MSPC-KA	1	IHC, MS
8	F	27	Healthy skin	1	IHC, MS
9	M	40	Healthy skin	1	IEM
10	M	68	Healthy skin	1	IF
11	F	44	Lymph node	1	IHC
12	M	23	Tonsil	1	IHC

IEM- immunoelectron microscopy; IET- immunoelectron tomography; IF – immunofluorescence; IHC-immunohistochemistry; KA- keratoacanthoma; MS- mass spectrometry; MSPC- multiple self-healing palmoplantar carcinoma.

Table S2. Primary antibodies used for immunolabeling

Antibody, isotype (ref, manufacturer)	Host	Dilution for IHC/IF	Dilution for IEM/IET
NLRP1, polyclonal IgG (ab36852, Abcam, UK)	Rabbit	1 µg/mL	1:25 (~0.04 µg/µL)
NLRP1, monoclonal IgG2b (MAB6788, Novus Biologicals, Germany)	Mouse	n/a	1:10 (~0.05 µg/µL)
Pro-IL1β/IL1β, polyclonal IgG (P420B, Invitrogen, Thermo Fisher Scientific, USA)	Rabbit	3.3 µg/mL	1:200 (~0.005 µg/µL)
NLRP3, recombinant monoclonal IgG (MA5-32255, Invitrogen, Thermo Fisher Scientific, USA)	Rabbit	n/a	1:25 (~0.04 µg/µL)
Isotype control, polyclonal IgG (ab37415, Abcam, UK)	Rabbit	Experiment dependent	Experiment dependent
Mast Cell Tryptase alpha/beta-1, monoclonal IgG1 kappa (clone AA1) (IR640, Dako, Agilent Technology, USA)	Mouse	Ready to use (used at 100 µL/section)	n/a
CD117/c-kit, polyclonal (A4502, Dako, Agilent Technology, USA)	Rabbit	1:400	n/a
IL18, polyclonal IgG (PA5-79479, Invitrogen, Thermo Fisher Scientific, USA)	Rabbit	1:200	n/a
TMS1/ASC [RM1049] (ab309497, Abcam, UK)	Rabbit	1:1200	n/a

IF- immunofluorescence; IHC - immunohistochemistry; IEM - immunoelectron microscopy; IET- immunoelectron tomography; n/a - not applicable

Video S1. 3D immunofluorescence showing colocalization of NLRP1 and tryptase in a skin mast cell. 3D-rendering of image stack with selective channel clipping, showing that NLRP1 (green) localizes to a mast cell from the skin of a healthy donor, and colocalizes with tryptase (red) inside cytoplasmic granules. Nucleus is stained with DAPI (blue).

Video S2. NLRP1 and NLRP3 colocalize to mast cell granules in MSPC-KA. In situ tomogram and segmentation showing colocalization of NLRP1 (magenta) and NLRP3 (green) to mast cell granules in MSPC-KA (related to Figure S3c,d).

Video S3. NLRP1, NLRP3 and IL1β colocalize to mast cell granules in MSPC-KA. In situ tomogram and segmentation showing colocalization of NLRP1 (magenta), NLRP3 (turquoise) and IL1β (green) to mast cell granules in MSPC-KA (related to Figure 4c-e).

Supplementary Figure S1. Additional immunohistochemistry controls for NLRP1 localization and for mast cell involvement in KA lesions. (a) Human lymph node stained against NLRP1, showing highest proportion of positive cells in the mantle zone of lymphoid follicles. Scale bar = 500 µm. Higher magnification **(inset**, scale bar = 20 µm**)** demonstrates localization of NLRP1 to granules of immune cells in lymph node. **(b)** Representative regions from non-MSPC KA, under different stains, showing absence of mast cell degranulation and epidermal infiltration. Scale bars are: 500 and 20 µm for (a) and inset, respectively; 1 mm for HE staining in (b) and 200 µm for all others panels in (b)

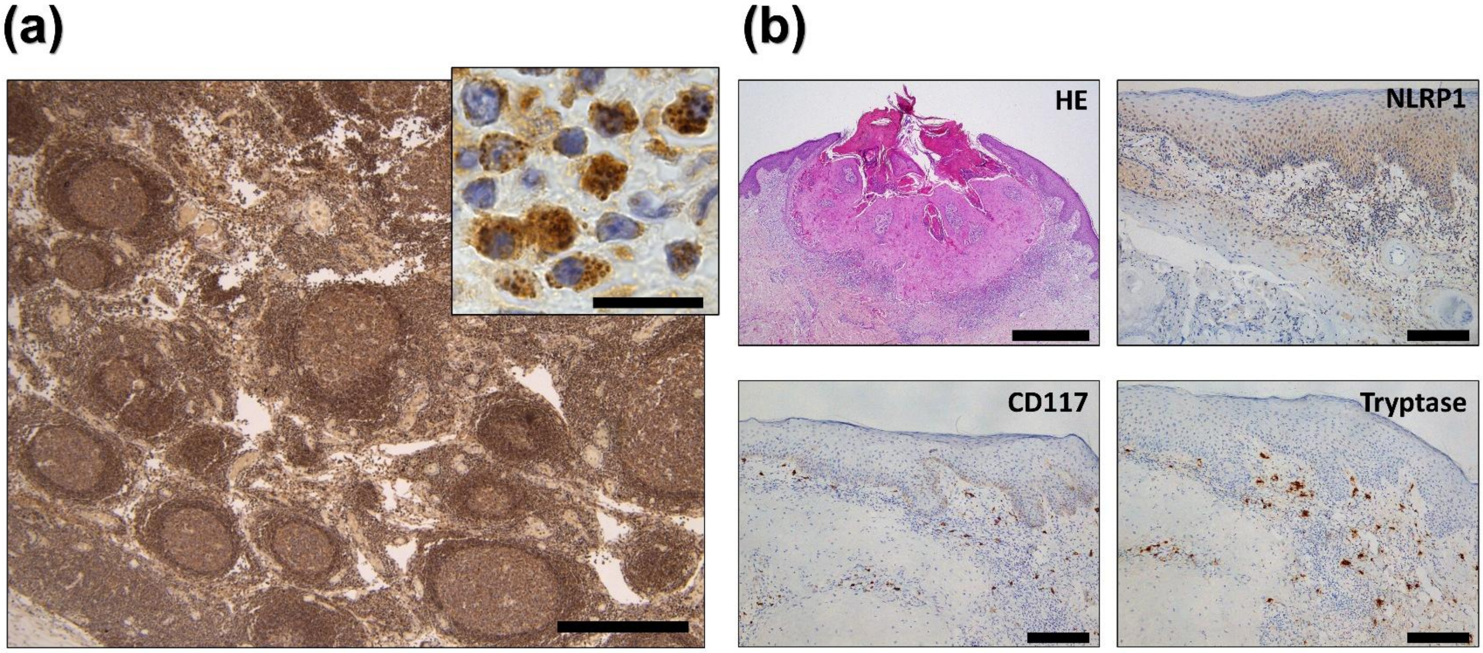

Supplementary Figure S2. Immunoelectron microscopy negative control and immunoelectron tomography of NLRP1 and NLRP3 colocalization in mast cell granules. (a) A rabbit isotype control did not significantly bind to mast cell (MC) granules. **(b)** NLRP1 localized to intermediate filaments (If) of keratinocytes. Representative accumulation of colloidal gold can be seen as black dots (circled area). *N - nucleus.* **(c)** Projection from tomographic tilt series, showing colocalization of 10 and 15 nm gold particles ($NLRP1^{A66V}$ and NLRP3, respectively) in granules from a MC in MSPC-KA. **(d)** Reconstructed tomogram with superimposed drawn contours of two MC granules (yellow and blue), presenting colocalization of $NLRP1^{A66V}$ (magenta) and NLRP3 (green). Detail of segmented MC granules demonstrating colocalization of $NLRP1^{A66V}$ (magenta) and NLRP3 (green) are presented in the boxed area. Scale bars = 500 nm for (a-b) and 300 nm for (c-d).

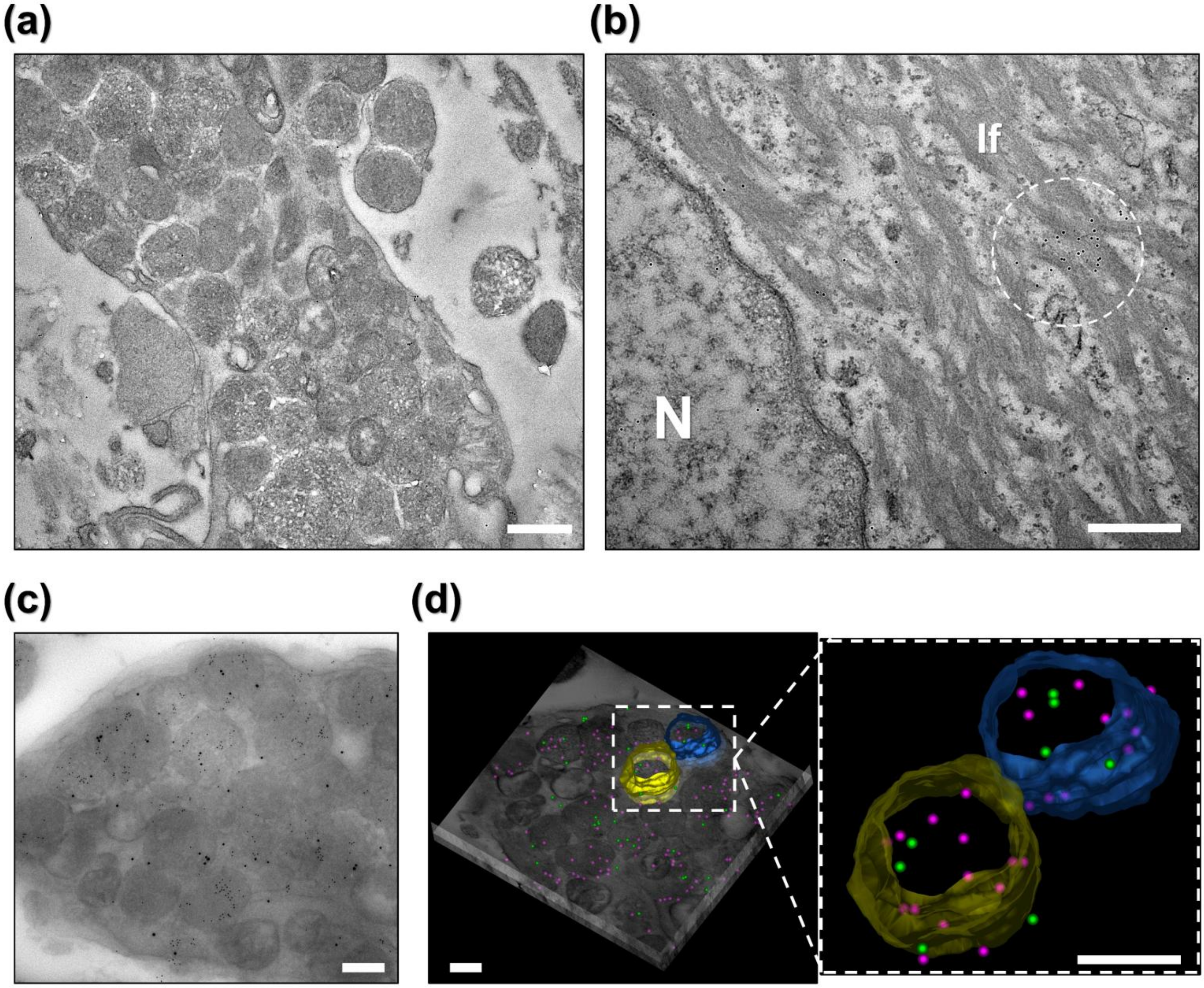

Supplement: Supplementary file 1 [file ijms-27-03775-s001.zip › Supplementary material.pdf]
